# DIagnostic Subdural EEG electrodes And Subdural hEmatoma (DISEASE): a study protocol for a prospective nonrandomized controlled trial

**DOI:** 10.1186/s42466-020-00096-8

**Published:** 2020-12-15

**Authors:** Sae-Yeon Won, Thomas M. Freiman, Philipp S. Reif, Daniel Dubinski, Elke Hattingen, Eva Herrmann, Volker Seifert, Felix Rosenow, Adam Strzelczyk, Juergen Konczalla

**Affiliations:** 1 Department of Neurosurgery, University Hospital, Goethe University Frankfurt, Schleusenweg 2-16, 60528 Frankfurt am Main, Germany; 2 Department of Neurology and Epilepsy Center Frankfurt Rhine-Main, University Hospital, Goethe-University Frankfurt, Frankfurt am Main, Germany; 3 LOEWE Center for Personalized Translational Epilepsy Research (CePTER), Goethe-University Frankfurt, Frankfurt am Main, Germany; 4 Institutes of Neuroradiology, Goethe University, Frankfurt, Germany; 5 Department of Medicine, Institute of Biostatistics and Mathematical Modelling, Goethe University, Frankfurt am Main, Germany

**Keywords:** Subdural hematoma, Seizure, Status epilepticus, Invasive EEG monitoring, Subdural strip electrode, Outcome

## Abstract

**Background:**

Epileptic seizures are common clinical features in patients with acute subdural hematoma (aSDH); however, diagnostic feasibility and therapeutic monitoring remain limited. Surface electroencephalography (EEG) is the major diagnostic tool for the detection of seizures but it might be not sensitive enough to detect all subclinical or nonconvulsive seizures or status epilepticus. Therefore, we have planned a clinical trial to evaluate a novel treatment modality by perioperatively implanting subdural EEG electrodes to diagnose seizures; we will then treat the seizures under therapeutic monitoring and analyze the clinical benefit.

**Methods:**

In a prospective nonrandomized trial, we aim to include 110 patients with aSDH. Only patients undergoing surgical removal of aSDH will be included; one arm will be treated according to the guidelines of the Brain Trauma Foundation, while the other arm will additionally receive a subdural grid electrode. The study’s primary outcome is the comparison of incidence of seizures and time-to-seizure between the interventional and control arms. Invasive therapeutic monitoring will guide treatment with antiseizure drugs (ASDs). The secondary outcome will be the functional outcome for both groups as assessed via the Glasgow Outcome Scale and modified Rankin Scale both at discharge and during 6 months of follow-up. The tertiary outcome will be the evaluation of chronic epilepsy within 2–4 years of follow-up.

**Discussion:**

The implantation of a subdural EEG grid electrode in patients with aSDH is expected to be effective in diagnosing seizures in a timely manner, facilitating treatment with ASDs and monitoring of treatment success. Moreover, the occurrence of epileptiform discharges prior to the manifestation of seizure patterns could be evaluated in order to identify high-risk patients who might benefit from prophylactic treatment with ASDs.

**Trial registration:**

ClinicalTrials.gov identifier no. NCT04211233.

## Background

Epileptic seizures are one of the frequent complications seen in patients with traumatic brain injury; the incidence is approximately 20% [1]. In particular, acute subdural hematoma (aSDH) is one of the most important predictors for seizures—alongside other parameters like age, preoperative Glasgow Coma Scale score, cerebral herniation, hematoma volume, and time to operation—associated with worse neurological outcome [2–8]. In a recent systematic review, the mean incidence of seizures in aSDH was 28%, whereas one retrospective study focusing on diagnostic electroencephalography (EEG) reported a very high incidence of epileptiform discharges on surface EEG scans in 87% of patients with aSDH. Thus, the question arises as to whether the incidence of seizures is underestimated [8, 9].

Despite the successful evacuation of subdural hematoma, approximately one third of patients show no clinical improvement without any medical explanation as to why. Surface spot EEG is routinely performed to detect seizures; however, the sensitivity of this approach is limited due to the skin–bone barrier and the short duration of recording. Furthermore, surface EEG is not always available as a diagnostic tool—for example, during the night or on weekends—which is an additional limitation leading to lengthier time to treatment. Spot surface EEG only records for 20 to 30 min in contrast with continuous EEG recordings which are performed for hours or days.

Due to the clinical relevance of seizures, several studies have investigated the benefit of prophylactic treatment with antiseizure drugs (ASD) [9, 10]. To date, there is only one recommendation from the Brain Trauma Foundation ruling evidence class II for treating patients with severe traumatic brain injury (TBI) with prophylactic ASD treatment during the first week based on data from Temkin et al. [11]. Beyond this time window, there was no clinical benefit for patients selected. Still, there are some limitations of the study in that the clinical use of prophylactic ASD treatment varies between clinicians and countries. In the 1980s, the standard medication was phenytoin, which has several side effects but by now several new intravenous antiepileptic drugs with comparable efficacy but better safety profiles have been introduced. Temkin et al. also did not distinguish between high-risk seizure-prone patients, like patients with aSDH, and low-risk patients, which is one of the limiting factors in supporting a more general recommendation on the treatment with ASDs. Therefore, the role of prophylactic ASD treatment is still questionable.

In the presurgical epilepsy evaluation, invasive EEG electrodes are commonly used to delineate the seizure-onset zone. The benefit of these electrodes relative to routine surface EEG is the possibility of real-time analysis in the case of seizure occurrence. It also enables direct monitoring of therapeutic effects. Therefore, the idea of this study was to make real-time analysis feasible in patients with TBI, particularly aSDH, to promote improved diagnostic and therapeutic real-time monitoring for detecting subclinical or nonconvulsive seizures.

### Study goals and objectives

Thus far, to our knowledge, no clinical study evaluating or monitoring seizures in patients with aSDH has been conducted. The surgical treatment of aSDH offers the unique opportunity to implant subdural EEG electrodes safely on sight and during the course of an already necessary surgical intervention, in order to demonstrate the potential benefits of invasive EEG in real-time, such as the earlier detection of seizures enabling faster therapeutic treatment and simultaneous therapeutic monitoring. The goal of the study is to evaluate the diagnostic and therapeutic effect of subdural invasive EEG monitoring to develop a standard treatment guideline for aSDH.

## Methods/design

### Trial design

**Fig. 1:**
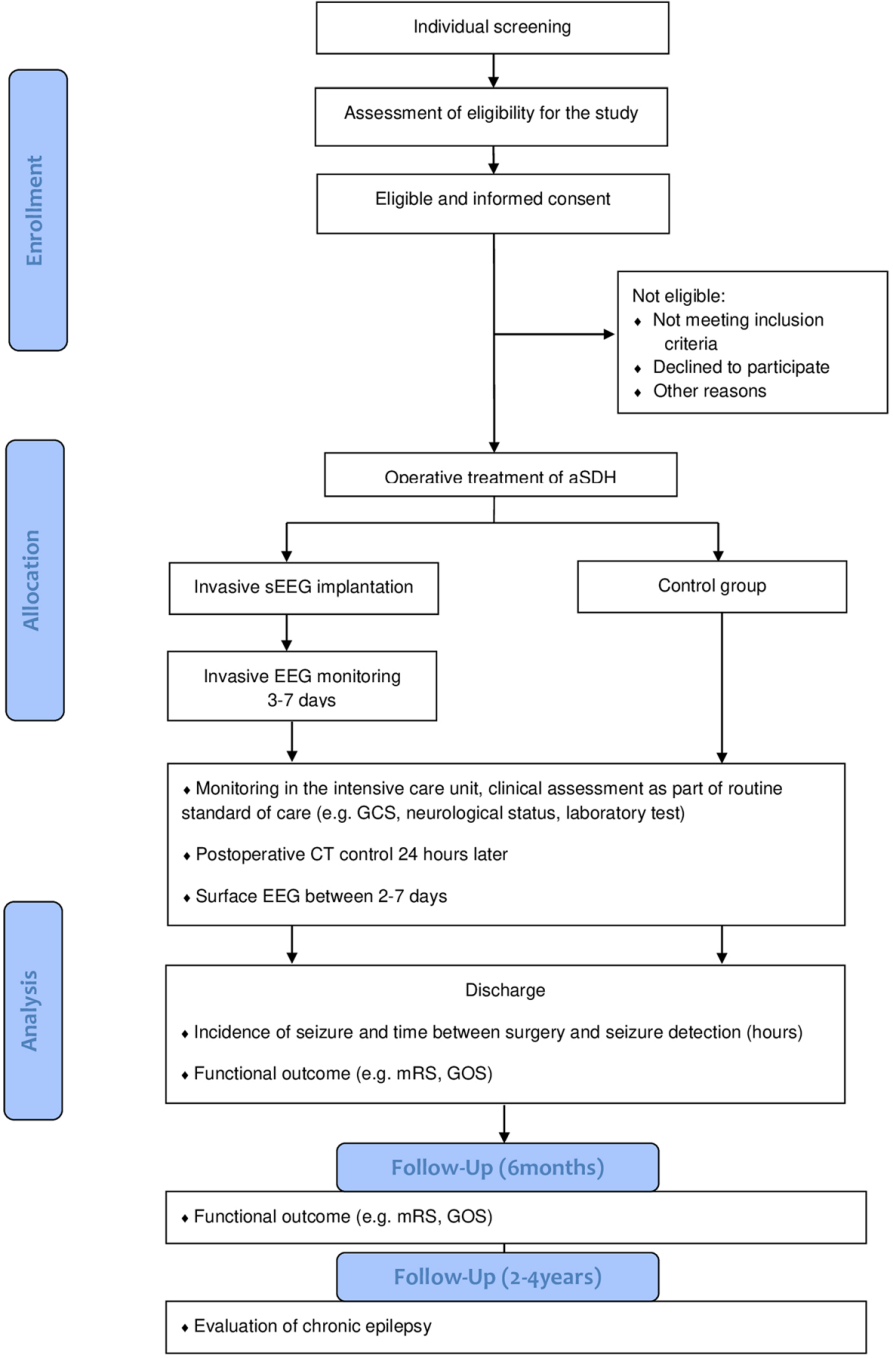
DISEASE study protocol flowchart

This is a prospective nonrandomized controlled study (phase I trial) where eligible participants will be divided into two arms: one intervention arm and one control arm. Enrollment, allocation, analysis and follow-up were summarized as a flow chart (Fig. 1).

### Study setting and inclusion/exclusion criteria

Patients will be recruited from the Department of Neurosurgery, University Hospital Frankfurt am Main following a diagnosis of aSDH. The local neurosurgical teams review patients upon admission and will assess eligibility for inclusion in the DISEASE trial. If informed consent is obtained and a mobile EEG system is available for recording, patients will be recruited into the intervention arm. Otherwise (e.g., a mobile EEG system is not available because it is already in use for the recording of other patients), patients will be placed in the control arm for follow-up.

This study will include adult patients (aged ≥18 years) with symptomatic aSDH requiring operative treatment via craniotomy or craniectomy who provided informed consent. Patients with infaust prognosis, asymptomatic patients with conservative treatment, those with aSDH as a secondary diagnosis, and those with concurrent enrollment in any other trial will be excluded.

### Informed consent

If the patient is able to consent, the neurosurgical staff member will explain the study design and further procedures to them. Written informed consent from the patient has to be obtained prior to the implantation of any invasive subdural electrodes. If the patient is unable to consent, a patient representative or independent physician may consent on behalf of the patient. Thereafter, if the patient’s clinical situation improves, consent should be sought from them at a later time.

### Trial interventions

The surgical procedure is standardized for both arms. All patients with aSDH will undergo the surgical evacuation of hematoma via craniotomy or craniectomy and receive subdural drain as well as an intracranial pressure monitoring probe. One arm would additionally receive a subdural EEG electrode with continuous monitoring (intervention arm), while the other arm would be treated with the standard treatment (control arm) according to the guideline from Brain Trauma Foundation as follow:

Treatment:

- Hyperventilation only as a temporizing measure to reduce ICP with maximum PaCo2 of 30 mmHg (evidence IIb)
- Barbiturate administration only to control elevated ICP refractory to maximum standard medical and surgical treatment (evidence IIb)
- Early tracheotomy in case of inadequate recovery (evidence IIa)
- Feeding patients to attain basal caloric replacement at least by the fifth day (evidence IIa)
- Low molecular weight heparin for the deep vein thrombosis prophylaxsis

Monitoring:

- Intracranial pressure monitoring (&lt;22 mmHg) (evidence IIb)
- Cerebral perfusion pressure monitoring (60-70 mmHg) (evidence IIb)
- Systolic blood pressure &gt;100 mmHg (evidence III)

**Fig. 2:**
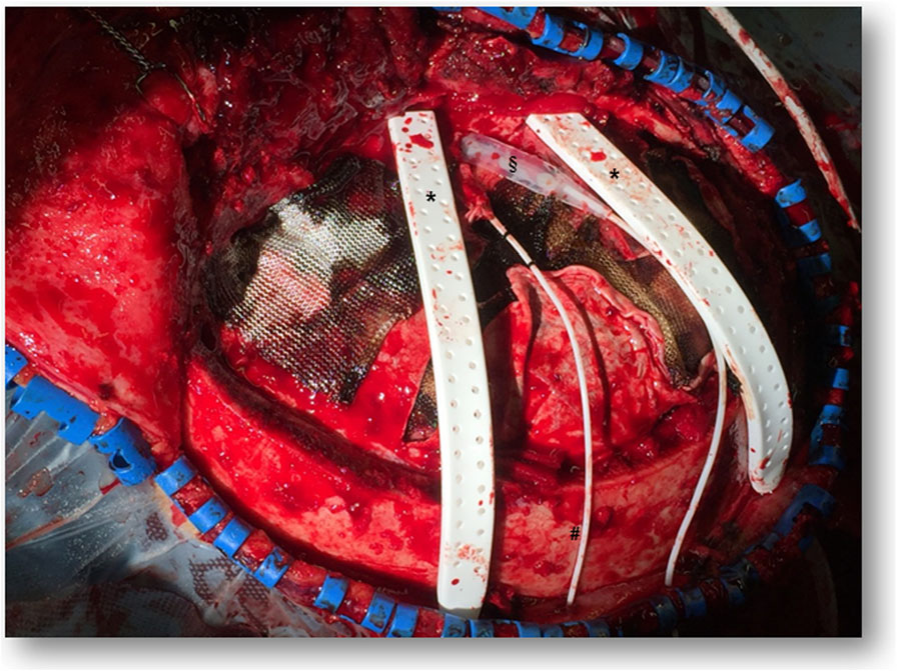
Intraoperative situs after the evacuation of acute subdural hematoma and insertion of two Jackson–Pratt drains (*), intracranial pressure probe (#) and frontotemporally placed subdural EEG electrode (§)

**Fig. 3:**
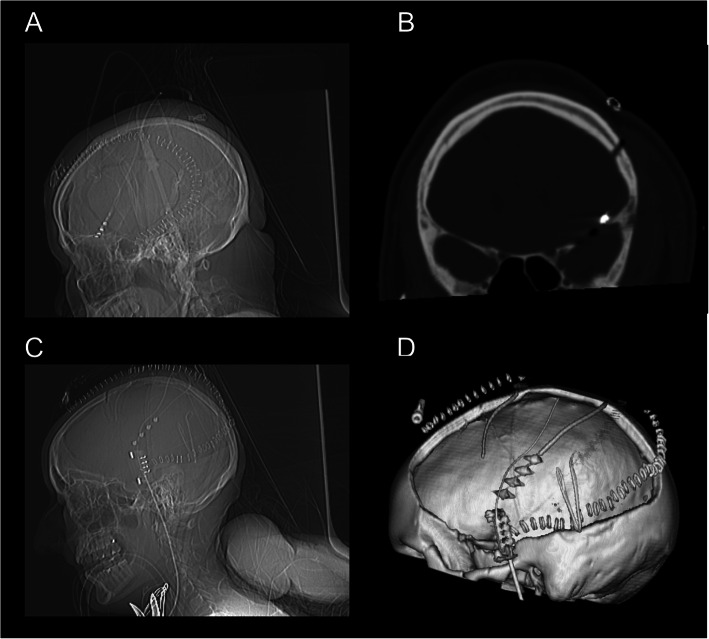
**a** Postoperative scout of CT scan with frontal EEG electrodes. **b** Coronary reconstruction of the CT scan. **c** Postoperative scout of CT scan with temporal EEG electrodes. **d** 3D reconstruction of the CT-scan

The subdural EEG electrode (PLATIN 1 × 4 or 1 × 6; Ad-Tech Medical Instrument Corporation, Oak Creek, WI, USA) will be intraoperatively implanted in the subdural space frontotemporally and diverted separately from the wound area (Fig. 2). Next, a postoperative CT scan will be performed and all patients will be monitored in the intensive care unit (Fig. 3).

**Fig. 4:**
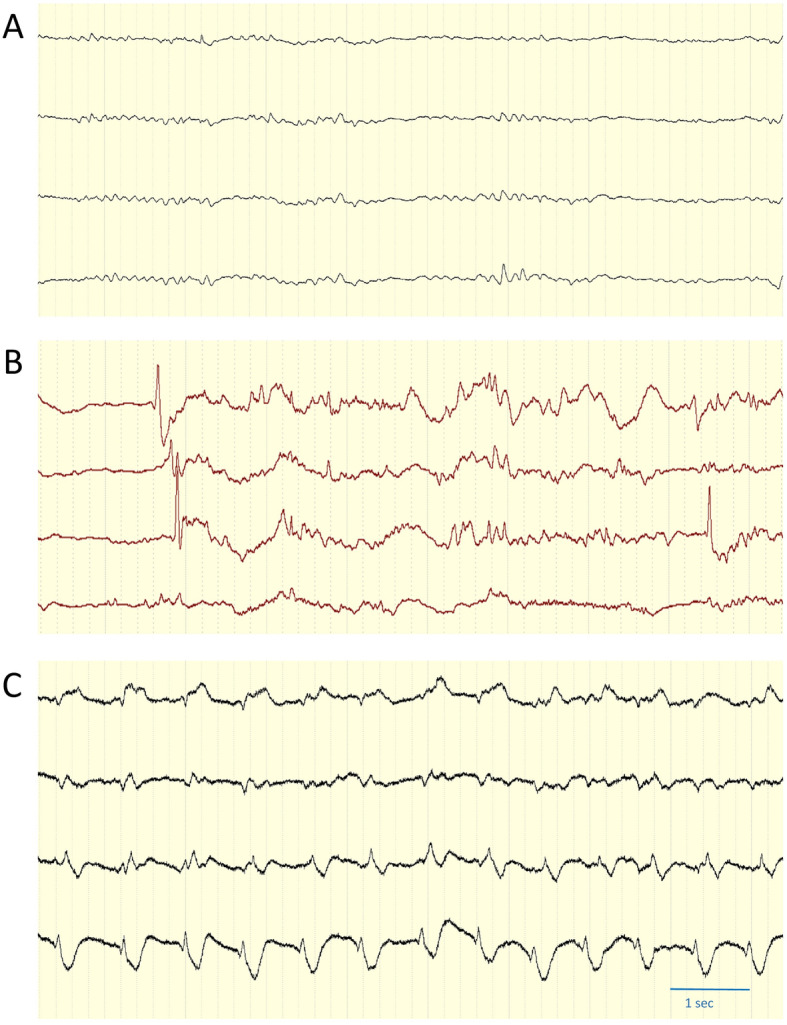
Subdural four-channel EEG recording showing diffuse slowing (**a**), epileptiform discharges (**b**) and a continuous seizure pattern (**c**)

As a clinical parameter, GCS will be evaluated every day and surface EEG will be performed at least once during the first 7 days post-operation in all patients. Additional surface EEGs will be performed if there is a suspicion of seizure. In the intervention arm, the continuation of invasive EEG monitoring will be evaluated by a board-certified epileptologist (Fig. 4). If a seizure is detected on EEG, treatment with benzodiazepines and ASDs will be started under further EEG monitoring and in consideration of the patient’s clinical condition. Treatment with ASDs will be adjusted on demand according to clinical and EEG assessment. If treatment with benzodiazepines and intravenous ASD is deemed insufficient for seizure suppression, escalation of treatment with anesthetics to obtain a seizure suppression or burst suppression pattern under EEG monitoring would be considered. The maximum period of invasive monitoring is set at 7 days, at which point the electrode should be removed. The subdural EEG electrode is removed simply by drafting as a normal wound drain and the incision sutured with Premilene 3.0 (Braun Melsungen AG OPM, D-34212 Melsungen, Germany).

### Definition of seizure

Seizure are defined as recommended by the International League Against Epilepsy, either as documented clinical seizure manifestation, ictal pattern in the EEG recordings, or clinical suspicion with interictal epileptiform discharges in the EEG recordings in temporal relationship with acute brain insult.

### Timeline

The anticipated timeline for study participants is as follows:

1. Patient admission with aSDH
2. Informed consent obtained (interventional arm/control arm)
3. Operative treatment of hematoma via craniotomy/craniectomy either with or without placement of subdural EEG electrode
4. Monitoring in intensive care unit
5. Postoperative CT control 24 h later
6. Clinical assessment as part of the routine standard of care (e.g., GCS, neurological status, routine laboratory tests)
7. Three to seven days of invasive EEG monitoring by a board-certified epileptologist
8. Optional spot surface EEG, usually between Days 1 and 7Removal of subdural EEG electrode at Day 7
9. Patient discharge after clinical evaluation
10. Outpatient clinical evaluation at 6 months postoperatively.
11. Evaluation of chronic epilepsy at follow-up in 2–4 years

### Trial outcomes measures

The primary and secondary outcome measures for this study are as follows:

Primary outcome measure:

1. Incidence of seizure and time between surgery and seizure detection

Secondary outcome measures

1. Functional and neurological outcomes (e.g., modified Rankin scale, Glasgow outcome scale) at discharge and for 3 to 6 months postoperatively
2. Therapeutic effects and benefits attributable to invasive monitoring and ensuing treatment (time to first interictal epileptiform discharges, occurrence of seizure patterns and progression of seizures to status epilepticus)
3. Complication rate of the intervention
4. Predictors for the development of early and late epileptic seizures
5. Longterm Follow-up for evaluation of chronic epilepsy (2–4 years)

All measures will be performed by the staff members of DISEASE-tiral. Independent from the study, all patients are scheduled for an outpatient clinic appointment in 6 months for clinical follow-up. Otherwise, the follow-up data will be retrieved by a phone call from the responsible study investigators. The long term follow-up will be performed accordingly.

### Clinical and morphological parameters

Following parameters will be collected in the prospective database: Basic characteristics (age, sex etc.), date of admission, timing of operation, timing of seizure, date of discharge, pupil status (isocor, anisocor, wide), operative treatment, comorbidities (arterial hypertension, cardiac disease,,respiratory disease, hematological disease, renal disease, infection, oncological disease etc.), anticoagulation, antiplatelet treatment, radiological parameters (volume of hematoma, midline shift, stroke,) GCS at admission/discharge/follow-up, GOS/mRS at discharge/follow-up and complications (bleeding, infection etc.).

### Adverse events

In a previous study by Johnston et al., the following complications of invasive subdural electrode monitoring in 112 children were described: wound infection (2.4%), cerebrospinal fluid leak (1.6%) and subdural hematoma, symptomatic pneumocephalus, bone flap osteomyelitis, and strip electrode fracture (each 0.8%). In contrast to this study, the complication rate in our study should be lower since we only used subdural strip electrode, that are smaller with limitation to four to six contacts, and less invasive than extended and multiple grid electrodes, furthermore the procedure will be performed only in patients with planned craniotomy/craniectomy [12].

In summary, serious adverse events will be recorded in the database and will include any of the following: intracerebral hemorrhage after removal of the electrode, infection or wound healing impairment requiring antibiotic treatment, death, and life-threatening events caused by the subdural EEG electrode. In these cases, the electrode will be removed and the observed adverse events will be reported to the local ethics committee.

### Statistical analysis and sample size

The IBM SPSS Statistics version 25 software program (IBM Corp., Armonk, NY, USA) will be used for data analysis. Data will be described using means ± standard deviations and numbers of patients, including percentages for continuous and categorical variables. For parametric parameters, an unpaired t-test will be used. For nonparametric parameters, variables will be analyzed in a contingency table using either Fisher’s exact test or the chi-squared test as appropriate. To assess the impact of the variables, odds ratios with 95% confidence intervals will be calculated. A *p*-value of 0.05 or less is considered to be statistically significant and all tests will be two-tailed.

When estimating the sample size with consideration of increased seizure detection by invasive EEG monitoring, the difference in the incidence rate is assumed to be 20%. The sample size was calculated as 110 patients (*n* = 55 patients each arm) to ensure 80% power (1-ß = 0.8). Moreover, time-to-event analysis will be performed to detect the timeline until seizure detection.

### Ethical issues

The study was approved by the local ethics committee (Goethe University Hospital, Frankfurt, EK 509/15). Moreover, the study is registered in the clinical study database ClinicalTrials.gov (NCT04211233).

### Study status

After approval of the study from the local ethics committee in the year 2016, a pilot study was performed to ensure the eligibility and safety of the study. Thereafter, the enrollment of patients was started and currently, several patients were recruited in the interventional and control arm. So far, there were no serious major complication observed, only one broken electrode was noticed without necessity of surgical revision. Figures 3 and 4 show findings in one patient with aSDH who underwent hemicraniectomy and hematoma evacuation. This patient was signed to the interventional arm and a subdural electrode with 4 contacts was implanted during the surgery (Fig. 3). At first postoperative day, the EEG signal showed flat patterns, whereas epileptiform discharges were first seen at postoperative day 2 and seizure patterns at postoperative day 3, treatment with ASDs was started (Fig. 4).

The subdural electrode was removed on day 7 without complications, the patient recovered well, and was discharged with mild neurological deficit.

## Discussion

Interest in subdural hematoma is currently rising, with lots of ongoing prospective studies being initiated [13–16]. While there are several new insights into the management and treatment of chronic subdural hematoma, there are less innovative developments regarding aSDH. Epileptic seizures and in particular status epilepticus are relevant complications of aSDH associated with poor quality of life and outcome [17–19]. Data on the real incidence of seizures and evidence-based recommendations regarding treatment strategies remain sparse. Given the limited capacity of surface EEG in the vast majority of hospitals, there is no developed tool widely available for accelerated diagnostic or therapeutic monitoring, leaving some clinicians to move forward with prophylactic treatment with ASDs, whereas other clinicians wait for the appearance of clinical seizure or status epilepticus prior to initiating treatment with ASDs. However, if a subclinical or nonconvulsive seizure can be detected early on, more timely treatment with ASDs might assist in stopping the ongoing seizure and avoiding unnecessary complications by prophylactic treatment. This can be managed by invasive subdural EEG monitoring. On the other hand, the use of subdural electrodes is associated with more potential complications like infection or subdural hematoma compared to surface EEG. Previously, a very low but still present complication rate of 0.85% has been reported [20]. A further limitation of subdural electrode application is the focal nature of its diagnostic power, which does not extend to the contralateral side. All in all, we think that the subdural electrode will be an inevitable mode of enrichment besides surface EEG.

Previously, several studies have been investigating the benefit of a continuous scalp EEG monitoring in patients with TBI [21, 22]. Struck et al. performed a prospective multicenter study analyzing over 5000 EEGs performed on over 4700 participants [21]. Hereby, 6 variables in EEG pattern could be identified creating a simple 2HELPS2B score system. Furthermore, this score system could be validated in other multicenter studies giving a robust basis for seizure prediction by EEG [21, 22]. We have no doubt that the continuous scalp EEG might be an inevitable future tool for seizure monitoring in TBI, still the invasive EEG has its advantage on the recorded hemisphere wherefore both monitoring could potentiate the sensitivity and specifity of seizure diagnostic. While preliminary results have been quite promising regarding detection rate and timing of seizure, questions of clinical benefit are still unanswered. This study expects to contribute to the validity of an important novel standard treatment for patients surgically treated for aSDH or traumatic brain injury in the future.

Potential limitations to this study include reaching an adequate recruitment number in the interventional arm, since aSDH is mostly an emergent situation where informed consent often cannot be obtained in a timely manner or at all. To overcome this limitation, an independent physician can also consent on behalf of a patient. Furthermore, the interventional and the control arm are not randomized due to the limited capacity of invasive, continuous EEG recording device. Therefore, there might be some bias in the selection criteria, however, we tried to overcome it by allocating every patient into interventional arm if the EEG recording device was available for use. Finally, this study is deployed only in a single center; however, after the phase I trial, we hope to conduct a multi-center prospective randomized trial to develop evidence-based recommendations for invasive subdural EEG monitoring in case of traumatic brain injury in general.

## Data Availability

The datasets used and/or analyzed during the current study are available from the corresponding author on reasonable request.

## Abbreviations

aSDH: Acute subdural hematoma
ASD: Antiseizure drugs
EEG: Electroencephalography
TBI: Traumatic brain injury
